# Epstein–Barr Virus DNA Exacerbates Arthritis in a Mouse Model via Toll-like Receptor 9

**DOI:** 10.3390/ijms25094661

**Published:** 2024-04-25

**Authors:** Nour Sherri, Rayan Assaf, Elio R. Bitar, Sabah Znait, Abdul Hamid Borghol, Aya Kassem, Elias A. Rahal

**Affiliations:** 1 Department of Experimental Pathology, Immunology, and Microbiology, American University of Beirut, Beirut 1107, Lebanon; nas51@mail.aub.edu (N.S.); ra448@aub.edu.lb (R.A.); erb07@mail.aub.edu (E.R.B.); sz60@aub.edu.lb (S.Z.); aib09@mail.aub.edu (A.H.B.); ak259@aub.edu.lb (A.K.); 2 Center for Infectious Diseases Research (CIDR), American University of Beirut, Beirut 1107, Lebanon

**Keywords:** EBV DNA, rheumatoid arthritis, Toll-like receptor 9, IL-17A, autoimmunity

## Abstract

Epstein–Barr virus (EBV) DNA is known to be shed upon reactivation of latent EBV. Based on our previous findings linking Toll-like receptor-9 (TLR9) to an EBV DNA-driven surge in IL-17A production, we aimed to examine the therapeutic potential of TLR9 inhibition in EBV DNA-exacerbated arthritis in a collagen-induced arthritis (CIA) mouse model. C57BL/6J mice were administered either collagen, EBV DNA + collagen, EBV DNA + collagen + TLR9 inhibitor, or only the TLR9 inhibitor. After 70 days, paw thicknesses, clinical scores, and gripping strength were recorded. Moreover, affected joints, footpads, and colons were histologically scored. Furthermore, the number of cells co-expressing IL-17A, IFN-γ, and FOXP3 in joint sections was determined by immunofluorescence assays. Significantly decreased paw thicknesses, clinical scores, and histological scores with a significantly increased gripping strength were observed in the group receiving EBV DNA + collagen + TLR9 inhibitor, compared to those receiving EBV DNA + collagen. Similarly, this group showed decreased IL-17A+ IFN-γ+, IL-17A+ FOXP3+, and IL-17A+ IFN-γ+ FOXP3+ foci counts in joints. We show that inhibiting TLR9 limits the exacerbation of arthritis induced by EBV DNA in a CIA mouse model, suggesting that TLR9 could be a potential therapeutic target for rheumatoid arthritis management in EBV-infected individuals.

## 1. Introduction

Epstein–Barr virus (EBV) or *Human herpes virus 4* (HHV4) is an enveloped double-stranded DNA virus that infects more than 90% of the population worldwide. EBV infection is typically asymptomatic in adults; however, it may cause infectious mononucleosis [1], a self-limiting disease that presents with fever, lymphadenopathy, and splenomegaly in adolescents [2]. EBV is suggested to be associated with various diseases, including lymphoid organ tumors, as well as autoimmune diseases such as multiple sclerosis (MS), systemic lupus erythematosus (SLE), and rheumatoid arthritis (RA) [3,4,5]. RA is a chronic inflammatory disease that affects the synovial membranes of several joints, causing joint pain and swelling. It is estimated that the prevalence of RA is 1% worldwide, and it is twice as common in women compared to men [6]. If the initiation of treatment is delayed, the chronic inflammation caused by RA may result in cartilage destruction, systemic manifestations, disability, and early death [7]. Like many autoimmune diseases, the exact cause of RA remains elusive. However, the onset of RA has been linked to a complex interplay of factors, encompassing genetic predisposition and environmental factors [8,9]. EBV infection has been suggested to be one of the most common environmental triggers that impacts the pathogenesis of RA [10,11].

Following its transmission via oropharyngeal secretions, EBV infects B-lymphocytes in the tonsils, where it establishes latency one to two weeks post-infection and could be reactivated in response to various exogenous signals [12,13]. This can result in recurrent infections during which viral antigens such as viral DNA are consistently shed. The persistent shedding of EBV DNA might activate pro-autoimmune responses and, therefore, play a role in the pathogenesis of autoimmune diseases such as RA [14,15]. Similarly, previous studies conducted by our group demonstrated that EBV DNA increases the production of IL-17A, a proinflammatory cytokine that has been extensively studied for its association with inflammation and autoimmune diseases, while the inhibition of Toll-like receptors (TLRs) 3, 7, and 9 significantly reduces the EBV DNA-mediated triggering of IL-17A production in mice [16,17]. Moreover, our group showed that EBV DNA increases the incidence and severity of arthritis in an RA mouse model (the collagen-induced arthritis (CIA) mouse model) [18]. Hence, targeting mediators that may recognize and respond to EBV DNA, such as endosomal TLRs, might serve as a potential prophylactic or therapeutic avenue that could benefit RA patients. Therefore, the aim of the study at hand is to assess whether the administration of a TLR9 inhibitor ameliorates the exacerbation of arthritis in an EBV DNA-injected RA mouse model. We evaluated the phenotypic manifestations of arthritis, the histological damage, and pro-inflammatory cellular foci in ankle joint tissues in EBV DNA-injected RA mice. Our objective is to identify a potential therapeutic tool for managing RA in EBV-infected individuals.

## 2. Results

### 2.1. Toll-like Receptor 9 (TLR9) Inhibition Attenuates the Phenotypic Manifestations of Arthritis in an Epstein-Barr Virus (EBV) DNA-Exacerbated RA Mouse Model

Based on our previous finding that EBV DNA increases the incidence and severity of arthritis in a CIA mouse model [18], we investigated the potential therapeutic effect of TLR9 inhibition on EBV DNA-exacerbated arthritis. At the end of the monitoring period, the severity of arthritis was assessed based on hind paw thickness, grip strength, and clinical scores of the affected paws.

**Figure 1:**
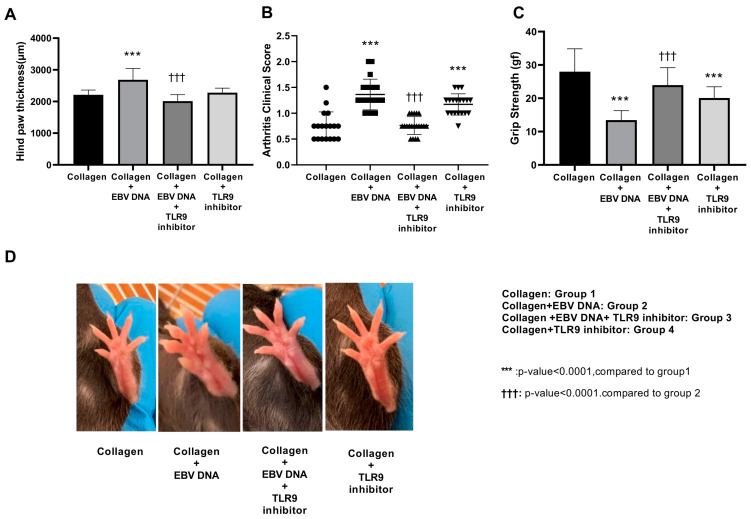
Effect of Toll-like receptor 9 (TLR9) inhibition on the phenotypic manifestations of arthritis in an Epstein-Barr virus (EBV) DNA-exacerbated rheumatoid arthritis (RA) mouse model. (**A**) Average hind paw thickness, (**B**) clinical scores of arthritis, and (**C**) average grip strength in C57BL/6J mice in groups 1, 2, 3, and 4. (**D**) Representative images of affected hind paws of arthritic mice in groups 1, 2, 3, and 4.

The average hind paw thickness was 2211.11 μm in group 1, 2685 μm in group 2 (*p* < 0.0001 compared to group 1), 2010 μm in group 3, (*p* < 0.0001 compared to group 2), and 2278.95 μm in group 4 (Figure 1A). The treatments of mice in groups 1, 2, 3, and 4 are described in the Section 4.

Consistent with the average hind paw thickness, group 1 exhibited moderate arthritis clinical scores, with approximately 88% of the scores falling below 1.25 (Figure 1B). However, group 2 showed significantly higher clinical scores in comparison with group 1 (*p* < 0.0001). Eighty percent of this group’s scores clustered at a score of 1.25 or above, suggesting an EBV DNA-driven exacerbation. On the other hand, group 3 demonstrated significantly lower clinical scores compared to group 2 (*p* < 0.0001). All scores of this group fell below 1.25, indicating a reduction in disease severity. In addition, the clinical scores of group 4 clustered below 1.5.

Joint inflammation in mice affects motor functions and results in a reduction in grip strength [19]. Hence, the grip strength in arthritic and TLR9 inhibitor-treated mice was measured (Figure 1C). The average grip strength in groups 1 and 4 was 27.87 gf and 20.7 gf, respectively. On the other hand, the grip strength significantly decreased (*p* < 0.0001 compared to group 1) in group 2, averaging 13.4 gf. In addition, group 3 showed a significant increase in the average grip strength (*p* < 0.0001) compared to group 2. The average grip strength of the latter group was 23.95 gf. Figure 1D demonstrates representative ankle joints from mice receiving different treatments. Individual mice from the different groups exhibited variability in the extent of redness and swelling.

### 2.2. TLR9 Inhibition Ameliorates the Arthritis Histological Scores in an EBV DNA-Exacerbated RA Mouse Model

**Figure 2:**
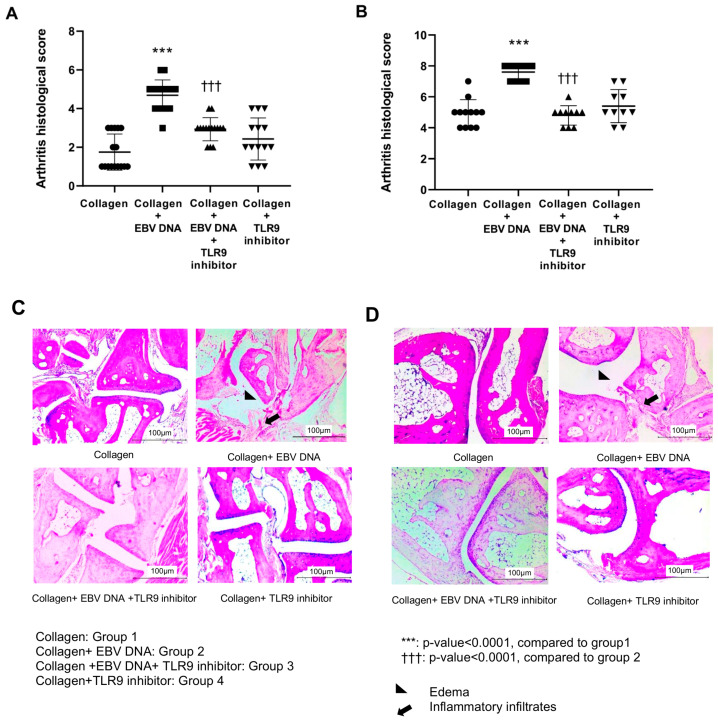
Effect of TLR9 inhibition on arthritis histological scores in an EBV DNA-exacerbated RA mouse model. (**A**) Histological scores of sections obtained from the footpads of C57BL/6J mice in groups 1, 2, 3, and 4. (**B**) Histological scores of sections prepared from the ankle joints of C57BL/6J mice in groups 1,2, 3, and 4. Representative histological sections of footpads (**C**) and ankle joints (**D**) obtained from the different groups of mice.

Histological examination was performed on the affected footpads (Figure 2A) and ankle joints (Figure 2B) to assess the effect of TLR9 inhibition on tissue damage and inflammation. In footpads, the histological scores for group 2 averaged to 4.68, which was significantly higher (*p* < 0.0001) than that of group 1, which averaged 1.75. However, the average histological score significantly decreased to 2.9 (*p* < 0.0001) in group 3 compared to group 1. In addition, the average histological score for group 4 was 2.4. 

Similar to the footpads, histological scoring of ankle joint tissue sections showed that group 2 had significantly (*p* < 0.0001) higher histological damage scores compared to group 1, with an average of 7.6, while that of the control group was 4.9 (Figure 2B). However, the average histological score of group 3 significantly decreased (*p* < 0.0001) compared to group 2. The average histological damage score for group 3 was 4.8. As for group 4, the average histological score was 5.4. Therefore, the administration of the TLR9 inhibitor resulted in a decrease in tissue damage and inflammation in EBV DNA-injected mice. Figure 2C,D show representative histological sections from footpads and ankle joints of arthritic mice belonging to different groups, respectively.

### 2.3. TLR9 Inhibition Ameliorates Colon Histological Damage in an EBV DNA-Exacerbated RA Mouse Model

**Figure 3:**
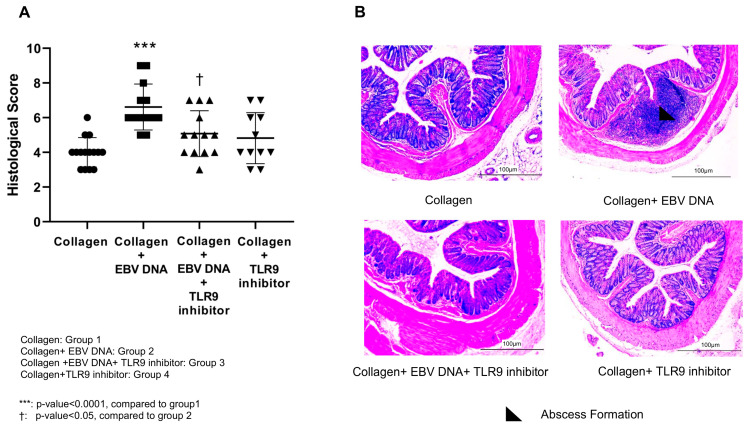
Effect of TLR9 inhibition on colon histological damage scores in an EBV DNA-exacerbated RA mouse model. (**A**) Histological damage scores of sections obtained from colons of C57BL/6J mice in groups 1, 2, 3, and 4. (**B**) Representative histological colon sections obtained from the arthritic mice.

Histological damage scoring of Hematoxylin and Eosin (H&E)-stained colon sections was performed based on inflammation severity, inflammation extent, and crypt damage (Figure 3A). In congruence with the histological scores of the footpads and ankle joints, group 2 had the highest damage score, averaging to 6.61, which was significantly (*p* < 0.0001) greater than that of mice in group 1, with an average of 3.53. A significant decrease (*p* = 0.01) was noticed in the histological score of group 3, attaining an average of 5.07. As for group 4, the histological score was 4.8. Figure 3B displays representative colon sections obtained from the different groups of arthritic mice. 

### 2.4. TLR9 Inhibition Decreases the Numbers of Double-Positive IL-17A+/IFN-γ+, Double-Positive IL-17A+/FOXP3+, and Triple-Positive IL-17A+/IFN-γ+/FOXP3+ Foci in an EBV DNA-Exacerbated RA Mouse Model

**Figure 4:**
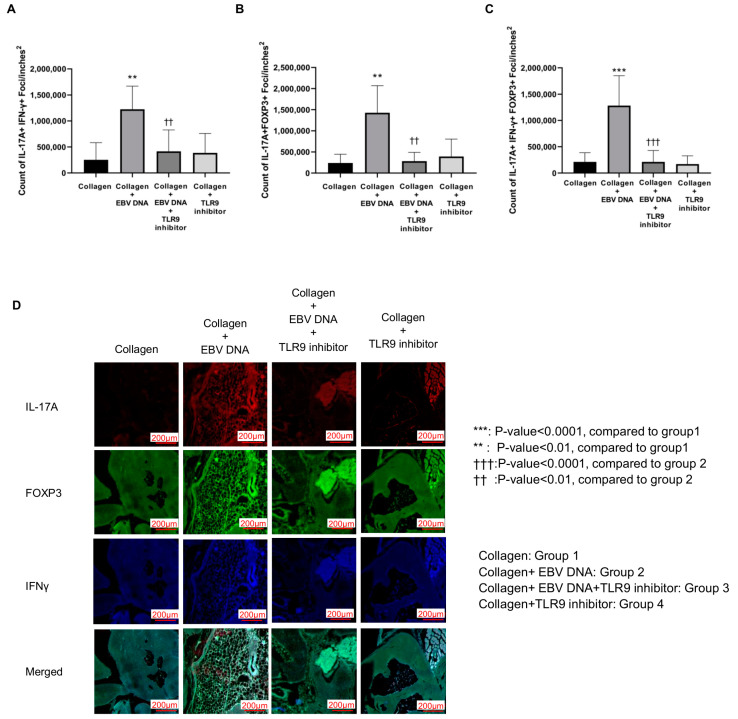
Effect of TLR9 inhibition on counts of local inflammatory immune cell populations in the joints of EBV DNA-injected arthritic mice. Counts of (**A**) IL-17A+ IFN-γ+, (**B**) IL-17A+ FOXP3+, and (**C**) IL-17A+ FOXP3+ IFN-γ+ foci in ankle joints of C57BL/6J mice in groups 1, 2, 3, and 4. (**D**) Immunofluorescence staining for IL-17A (red), IFN-γ (blue), and FOXP3 (green) in the ankle joints of experimental and control mouse groups.

To assess the potential therapeutic effect of TLR9 inhibition on decreasing the local inflammation in the joints of EBV DNA-injected arthritic mice, histological sections from ankle joints were immunostained for the following markers: IL-17A, IFN-γ, and FOXP3. Using confocal microscopy, the number of foci that were triple-positive IL-17A+/IFN-γ+/FOXP3+, double-positive IL-17A+/IFN-γ+, and double-positive IL-17A+/FOXP3+ were counted. The double-positive IL-17A+/IFN-γ+ counts were the highest in group 2, with a significant increase in counts (*p* = 0.0019) when compared to group 1. On the other hand, group 3 had significantly lower counts (*p* = 0.0096) when compared to group 2 (Figure 4A). Similarly, group 2 exhibited the highest numbers of double-positive IL-17A+/FOXP3+ counts, which was significantly higher (*p* = 0.0003) than that of group 1. The double-positive IL-17A+/FOXP3+ counts in group 3 significantly decreased (*p* = 0.0005) compared to group 2 (Figure 4B). As for the triple-positive IL-17A+/IFN-γ+/FOXP3+ counts (Figure 4C), these were the highest in group 2, with a significant difference (*p* < 0.0001) when compared to group 1. However, these counts were significantly lower (*p* < 0.0001) in group 3 when compared to group 2. Figure 4D displays immunofluorescence staining for the markers IL-17A, IFN-γ, and FOXP3 in all mouse groups.

## 3. Discussion

Infectious agents, including viruses, are among the most significant environmental factors that may trigger an abnormal immune response, resulting in the development of autoimmune diseases such as RA. EBV DNA that is shed during the reactivation of an EBV infection increases the production of pro-inflammatory cytokines and hence contributes to the pathogenesis of autoimmune diseases. In congruence, previous studies conducted by our group demonstrated that the intraperitoneal administration of EBV DNA in mice increases the production of IL-17A, reaching its peak 6 days post-injection. This surge in IL-17A production was found to be significantly reduced by the inhibition of endosomal TLRs, particularly TLR9 [16,17]. Moreover, our group has demonstrated that EBV DNA increases the incidence and severity of arthritis when administered in a CIA mouse model [18]. Knowing that TLR9 recognizes unmethylated CpG motifs, which are abundantly found in EBV DNA, and based on our aforementioned findings, we aimed in this study to investigate the potential therapeutic effect of TLR9 inhibition on the severity of EBV DNA-exacerbated arthritis in an RA mouse model.

In accordance with findings documented in a previous study conducted by our team, group 2, which received EBV DNA in addition to collagen, had a significantly higher clinical score and hind paw thickness average than group 1, which only received collagen [18]; this suggests that EBV DNA worsens the clinical symptoms of arthritis in the mouse model of the disease. Interestingly, a significantly lower clinical score and hind paw thickness average were observed in group 3, which had additionally received an intraperitoneal injection of the TLR9 inhibitor along with EBV DNA and collagen. This finding indicates that TLR9 inhibition ameliorates the phenotypic manifestations of the disease.

As for the average grip strength, which is measured to assess the motor functions of the affected paws in mice, it was significantly lower in group 2 in comparison to group 1, further demonstrating that EBV DNA worsens arthritis in the RA mouse model. Moreover, the average grip strength was significantly higher in group 3 than it was in group 2. This demonstrates that TLR9 inhibition reduces the burden and the clinical manifestations of the disease in EBV DNA-injected arthritic mice.

In line with the clinical evaluation, the histological damage score of the affected footpads and ankle joints was significantly higher in group 2 than in group 1, demonstrating that EBV DNA exacerbates the clinical symptoms of RA in the mouse model. On the other hand, group 3 had a significantly lower histological damage score than group 2, which is consistent with both the clinical score and grip strength results. This indicates that TLR9 inhibition reduces tissue damage and inflammation in EBV DNA-injected arthritic mice.

RA is a systemic inflammatory disorder that might affect extra-articular organs, including the colon [20]. Therefore, we evaluated the effect of TLR9 inhibition on histological damage in the colons of EBV DNA-injected arthritic mice. Consistent with the clinical evaluation and the histological scoring of the footpads and ankle joints, group 2 exhibited a significantly higher histological damage score compared to group 1. Furthermore, group 3 had a significantly lower histological score compared to group 2. These results suggest that EBV DNA not only induces local inflammatory damage in the joints of arthritic mice but may also induce inflammation in extra-articular regions. In addition, the results indicated that TLR9 inhibition may mitigate the systemic inflammation induced by EBV DNA in arthritic mice.

Although TLR9 has been suggested to play a role in the pathogenesis of RA through enhancing T-cell-mediated inflammation and being involved in osteoclast formation [21], our findings did not indicate a therapeutic effect of TLR9 inhibition in arthritic mice not receiving an EBV DNA injection. On the contrary, this group displayed more severe clinical manifestations of RA than the group that received collagen only. In congruence with our results, a previous study conducted by our group showed that BALB/c mice receiving only ODN2088, the TLR9 inhibitor employed in our study, exhibited an increase in their IL-17A levels [16]. Furthermore, another study indicated that ODN2088 enhances IL-17A secretion in human helper T cells [22]. These observations could be attributed to possible crosstalk occurring between the TLR9 signaling pathway and other immune pathways triggered by various DNA sensors. Due to the complexity and interconnection of these pathways, the inhibition of one may possibly lead to eliciting a compensatory activity through another.

Multiple studies have previously demonstrated that IL-17A plays a crucial role in the pathogenesis of RA, whereby it is implicated in pannus development, matrix degradation, cartilage destruction, and osteoclast differentiation [23]. On the other hand, the role of IFN-γ in RA is multifaceted, involving both pathogenic and regulatory functions [24]. Immune cells co-expressing IL-17A and IFN-γ are found in higher numbers in inflammatory conditions, and they are thought to be involved in the pathogenesis of multiple autoimmune diseases [25,26]. Moreover, although FOXP3 is known to be a marker of regulatory T cells (Tregs), which are responsible for regulating inflammation, a study revealed a higher count of IL-17A+ FOXP3+ cells in the blood of RA patients compared to healthy controls. Similar to Th17 cells, these FOXP3+ cells can secrete IL-17, IL-22, and IL-21 [27]. Additionally, several studies have indicated that IL-17A+ FOXP3+ cells do not only produce IL-17A but also IFN-γ and IL-2 [28,29,30]. This suggests a potential mechanism where defective Treg function could lead to ongoing autoimmunity, characterized by the loss of FOXP3 expression under inflammatory conditions and induction of proinflammatory cytokines such as IL-17A and IFN-γ (IFN-γ+ FOXP3+ IL-17A+) [31,32,33].

In our study, group 2 had the highest count of IL-17A+/IFN-γ+/FOXP3+, IL-17A+/IFN-γ+, and IL-17A+/FOXP3+ immune cells compared to group 1. These mice also exhibited the most severe symptoms, which is consistent with the earlier observations. However, the count of IL-17A+/IFN-γ+/FOXP3+, IL-17A+/IFN-γ+, and IL-17A+/FOXP3+ cells was significantly lower in group 3 compared to group 2. This suggests that TLR9 inhibition suppresses inflammation and subsequently curbs the differentiation of T cells into pathogenic inflammatory immune cell populations involved in the progression of RA.

While our findings may potentially pave the way for improved management of RA in EBV-infected individuals, it is important to view them in the context of certain limitations. The adopted mouse model, the CIA mouse model, shares several key characteristics with human RA, such as the loss of immune tolerance and the production of autoantibodies targeting collagen [34]. On the other hand, RA is a complex autoimmune disease that encompasses various pathogenic mechanisms and is not limited to the inflammation accompanied by the production of autoantibodies against collagen. Moreover, although mice and humans possess numerous shared genes and exhibit comparable immune systems, the processes governing immune system development and activation diverge between the two species. For instance, the structure of TLR9 in humans differs from that of mice by approximately 24% [35]. Such structural differences might affect ligand binding and subsequently alter the outcome of downstream signaling pathways. In addition to TLR9, EBV DNA may be recognized by numerous nucleic acid-sensing receptors that activate downstream signaling pathways, enhancing inflammatory responses. Therefore, future studies investigating the implication of these receptors in the EBV DNA-driven exacerbation of RA should be conducted. Moreover, since EBV infection is correlated with the development of multiple autoimmune diseases, it might be useful to examine the effect of TLR9 inhibition on the progression and severity of other autoimmune diseases associated with EBV infection.

In conclusion, our study demonstrates that TLR9 inhibition ameliorates the outcome of EBV DNA-exacerbated arthritis in an RA mouse model. Given the high prevalence of EBV and its ability to establish lifelong persistent infections with the potential for reactivation, TLR9 inhibitors may serve as a potential therapeutic tool to be considered for RA management in EBV-infected individuals.

## 4. Materials and Methods

### 4.1. EBV DNA Preparation

P3HR-1, a Burkitt’s lymphoma cell line latently infected with the EBV type 2 strain, was employed (The American Type Culture Collection (ATCC), Rockville, MD, USA). They were cultured in Roswell Park Memorial Institute (RPMI) 1640 culture medium (Lonza, Basel, Switzerland) supplemented with 20% fetal bovine serum (FBS) (Sigma-Aldrich, Saint Louis, MO, USA) and 1% penicillin-streptomycin (PS) from Lonza. The cells were cultured at 37 °C with 5% CO_2_ and passaged on a regular basis, sustaining them at 70% confluency.

To induce EBV replication, 65 ng/mL of phorbol 12-myristate 13-acetate (PMA) (Sigma-Aldrich, Saint Louis, MO, USA) was added to the P3HR1 cells. After 5 days, the cells were centrifuged at room temperature for eight minutes at 800 rpm to obtain the virus-containing supernatant. Then, to pellet the virus, the supernatant was centrifuged once more for 90 min at 16,000× *g* at 4 °C. 

A 100 μL volume of the viral preparation was mixed with an equivalent of Tris-HCL-saturated phenol (pH 6.7–7.9) with the intent to lyse the virus and extract the DNA. Following a brief vortex step, the sample was centrifuged at 13,000 rpm for 15 min at 4 °C. The genomic DNA-containing upper aqueous layer was collected and mixed with 3 M cold sodium acetate (pH = 5.2) and cold 100% ethanol (Sigma-Aldrich). To precipitate the DNA, the samples were left at −80 °C overnight. The samples were centrifuged the next day to collect the DNA pellet for 15 min at a speed of 13,000 rpm at 4 °C. Then, the pellet was washed twice, with each wash consisting of adding 1 mL of 70% ethanol, centrifuging at 13,000 rpm for 15 min at 4 °C, and discarding the supernatant. Finally, the DNA pellet was air-dried before being resuspended in 30 μL of nuclease-free water.

To quantify the total number of EBV DNA copies, real-time PCR was performed. EBV DNA copies were quantified using Taq Universal SYBR Green Supermix (Bio-Rad, Berkeley, CA, USA) on a Bio-Rad CFX96TM Real Time PCR Detection System. The EBV-encoded small RNA 2 (*EBER-2*) primers used were obtained from Macrogen (Seoul, Republic of Korea) (Table 1).

In a total volume of 10 μL, the real-time PCR reactions contained 4 μL SYBR green, 1 μL forward primers and 1 μL reverse primers (7.5 pmol/μL), and a 1 μL sample DNA. The Bio-Rad CFX96/TM system’s thermal cycling program was set to perform three steps: a 5 min initial activation stage at 95 °C, followed by 40 cycles at 95 °C for 15 s, and finally, a 58 °C annealing stage for 30 s.

The standard curve was established by varying the number of copies of the EBV DNA control in each reaction (1000, 2000, 5000, 10,000, 54,000) (Amplirun Epstein Barr Virus DNA Control, 32 Vircell S.L., Granada, Spain). The standard curve was considered to be acceptable when the slope ranged from −3.0 to −3.6, and the correlation coefficient was at a minimum of 0.98.

### 4.2. Mice

CIA in C57BL/6J mice was the adopted murine arthritis model. Female mice between 9 and 12 weeks old were utilized. The American University of Beirut (AUB) animal care center provided the mice, which were then handled in accordance with the Institutional Animal Care and Use Committee (IACUC) regulations. They were co-housed in a room with a temperature of 22–25 °C and a 12 h light–dark cycle in non-individually ventilated cages (non-IVC). They had unrestricted access to food and water. To achieve randomized assignment, mice were given different group labels and randomly placed ear tags.

### 4.3. Induction of Arthritis in C57BL/6 Mice and Treatment Regimen

To establish the CIA mouse model in female C57BL/6 mice, an arthritis-inducing emulsion was prepared as previously demonstrated [36,37]. The emulsion was created by mixing equal parts of complete Freund’s adjuvant (CFA) and type II chicken collagen (Chondrex Inc., Redmond, WA, USA). CFA was prepared by combining incomplete Freund’s adjuvant (IFA) (Chondrex Inc., Redmond, WA, USA) with 3.3 mg/mL of heat-killed Mycobacterium tuberculosis (Invivogen, Toulouse, France). To increase the incidence of arthritis in mice, Type II chicken collagen booster injections were given 20 days after the primary immunization. The booster emulsions were prepared by combining type II chicken collagen with IFA instead of CFA. The mice were anesthetized using sevoflurane inhalation, and then 50 μL of type II chicken collagen/adjuvants mixture was administered subcutaneously in the tail. 

**Figure 5:**
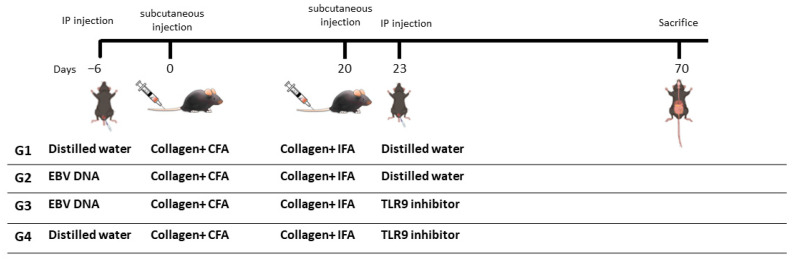
Treatment regimen for assessing the effect of TLR9 inhibition on EBV DNA-exacerbated RA in type II chicken collagen-induced arthritis (CIA), C57BL/6J mouse model. EBV, Epstein-Barr virus; CFA, complete Freund’s adjuvant; IFA, incomplete Freund’s adjuvant; IP, intraperitoneal.

To assess the effect of TLR9 inhibition on the severity of EBV DNA-exacerbated arthritis, mice were divided into four groups of 20 (Figure 5). The first group (group 1) received type II chicken collagen in CFA to induce RA (primary immunization—day 0). This group served as an arthritis control group. The second group (group 2) received 144 × 10^3^ copies of EBV DNA 6 days prior to the primary immunization in addition to the type II chicken collagen that will be given at the beginning of the experiment (day 0). The selected time point for EBV DNA injection is based on our previous observation that the incidence of arthritis was significantly higher and occurred within a narrower period of time when EBV DNA was administered 6 days prior to the initial collagen challenge [18]. The third group (group 3), which served as the treatment group, received 144 × 10^3^ copies of EBV DNA 6 days prior to the primary immunization in addition to a TLR9 inhibitor (Macrogen) 23 days after the primary immunization. Moreover, the fourth group (group 4) received a TLR9 inhibitor 23 days after the primary immunization. All mouse groups received a booster immunization after the primary immunization. EBV DNA and TLR9 inhibitor were administered intraperitoneally (IP). The injected dose of the TLR9 inhibitor included in Table 2 is based on our previous finding that this dose decreases IL-17A levels when administered in EBV DNA-injected mice [16]. The mice were then monitored for clinical signs of arthritis development after the primary immunization for a period of 70 days.

### 4.4. Evaluation of TLR9 Inhibitor Impact on the Severity of Arthritis in EBV DNA-Exacerbated RA Mouse Model

The severity of arthritis was assessed by measuring hind paw thickness and clinically scoring the affected hind paw of the mice. The thickness of each mouse’s hind paws was measured using a caliper. Moreover, to determine the clinical score, each affected paw was checked for redness and swelling according to the scoring system shown in Table 3, which was modified from a previous study [38]. If more than one paw was affected in one mouse, the final score was calculated by averaging the sum of the values obtained. To assess the motor function of the affected joints, a grip strength test was performed at the end of the monitoring period using a grip strength meter (Ugo Basile, Gemonio (VA), Italy). Each mouse was gently lifted by the tail such that its affected paw could grasp onto the steel grip. The mouse was then pulled until it lost its grip. The peak gram force (gf) required to make the mouse release its grip was recorded and evaluated in triplicate to determine the average for each mouse. Moreover, a histopathological evaluation of the affected ankle joints, footpads, and colons was performed. Footpads and joints were decalcified with Protocol TM Decalcifier B (Thermo Fisher Scientific, Waltham, MA, USA) and embedded in paraffin after being fixed in 10% formaldehyde. The sections were then stained with Hematoxylin and Eosin (H&E) and scored based on different pathological factors. The Scoring systems adopted to histologically score the footpads and ankle joint sections are detailed in Table 4 and Table 5, respectively. Likewise, the dissected colons were fixed using 10% formaldehyde, embedded in paraffin, and stained using H&E. After preparing the histological sections, histological scoring of the colons was performed based on inflammation severity, inflammation extent, and crypt damage (Table 6). Blinded scoring was conducted by two blinded independent scorers. The scores of the different parameters were summed up.

### 4.5. Assessment of Potential Therapeutic Effects of TLR9 Inhibition on Decreasing Local Inflammation in the Joints of EBV DNA-Injected Arthritic Mice

Immunofluorescence staining was performed on ankle joint histological sections to count the number of cells that were IL-17A+/IFN-γ+/FOXP3+, IL-17A+/IFN-γ+, or IL-17A+/FOXP3+ in the different groups of mice. After being heated at 55 °C for 40 min, the unstained section slides were deparaffinized by being immersed into xylol twice for ten minutes each. The sections were then rehydrated by submerging the slides in ethanol solutions with progressively lower concentrations (100%, 95%, and 75%) for five minutes each. Following the deparaffinization procedure, the slides were submerged in distilled water for 5 min twice. To perform antigen retrieval, the slides were then submerged in citrate buffer (pH 6) for 90 min in a water bath that was heated to 60 °C. Tri-sodium citrate dihydrate (0.1 M) and citric acid (0.1 M) were combined to create the citrate buffer (18 mL of citric acid, 82 mL of tri-sodium citrate dehydrate, and 900 mL of distilled water). The slides were removed from the water bath, allowed to cool in the citrate buffer for 30 min, and finally went through two rounds of washing in which they were immersed in distilled water for 5 min each time. The slides were then rinsed three times with 1× PBS to remove the permeabilization buffer after being permeabilized for five minutes at room temperature in Tris-buffered saline (TBS; pH 7.4). For TBS preparation, 3% Triton X and 1× PBS were mixed. The slides were incubated in the blocking solution (15% FBS in 1× PBS) for 60 min at room temperature. Then, an antibody dilution buffer was prepared using 1× PBS, 15% FBS, and 0.3% Triton X. Fluorochrome-linked primary antibodies were then incubated on the slides overnight. Fluorochrome-linked Brilliant Violet 605 anti-mouse IL-17A (1:1500), Pacific Blue 405 anti-mouse IFN (1:1500), and Alexa Fluor 488 anti-mouse FOXP3 (1:1500) antibodies were employed (Biolegend, San Diego, CA, USA). After being washed twice with 1× PBS, the slides were covered with a mounting solution (80% glycerol, 223 mM 1,4-diazabicyclo [2.2.2] octane (DABCO), and 4 mM Tris-HCl) and a coverslip before being stored at 4 °C. The slides were examined with a laser scanning confocal microscope (Zeiss, Oberkochen, Germany) 57 and the Zen 2.3 SP1 software.

### 4.6. Statistical Analysis

Graphpad Prism v6 was employed for statistical analysis. The Mann–Whitney U Test was used to compare histological and clinical scores. One-way and Tukey tests were used to compare means. *p*-values less than 0.05 represented statistical significance.

## Figures and Tables

**Table 1:** Primer sequences.

Primer	Sequences
*EBER-2*	F: 5′-CCCTAGTGGTTTCGGACACA-3′
	R: 5′-ACTTGCAAATGCTCTAGGCG-3′

**Table 2:** TLR9 specifications.

TLR9	Diluent	Dose per Volume
ODN2088	Distilled water	56 μg in 100 μL

**Table 3:** The clinical scoring system adopted for assessment of arthritis in C57BL/6J mice.

Clinical Observation	Arthritis Clinical Score
No redness and swelling	0
Slight redness	0.25
Slight redness and swelling	0.5
Mild redness and swelling	0.75–1
Moderate redness and swelling	1.25–1.5
Severe redness and swelling	1.75–2

**Table 4:** Histological scoring system for footpad evaluation.

Footpad Joint Section Histological Scoring		
Inflammatory Infiltrates	Edema	Histological Score
None	None	0
Mild	Mild	1
Moderate	Moderate	2
Severe	Severe	3

**Table 5:** Histological scoring system for ankle joint evaluation.

Ankle Joint Section Histological Scoring				
Inflammatory Infiltrates	Edema	Cartilage Destruction	Connective Tissue Disruption	Histological Score
None	None	None	None	0
Mild	Mild	Mild	Mild	1
Moderate	Moderate	Moderate	Moderate	2
Severe	Severe	Severe	Severe	3

**Table 6:** Histological scoring system for colon evaluation.

Colon Section Histological Scoring			
Inflammation Severity	Inflammation Extent	Crypt Damage	Histological Score
None	None	None	0
Mild	Mucosa	1/3 Basal Damage	1
Moderate	Submucosa	2/3 Basal Damage	2
Severe	Transmural	Loss with present surface epithelium	3
		Crypt loss and surface epithelium loss	4

## Data Availability

Data are available upon request submitted to the corresponding author.
